# Amino-Termination of Silicon Carbide Nanoparticles

**DOI:** 10.3390/nano13131953

**Published:** 2023-06-27

**Authors:** Szabolcs Czene, Nikoletta Jegenyes, Olga Krafcsik, Sándor Lenk, Zsolt Czigány, Gábor Bortel, Katalin Kamarás, János Rohonczy, David Beke, Adam Gali

**Affiliations:** 1 Doctoral School on Materials Sciences and Technologies, Óbuda University, Bécsi út 96/b, H-1034 Budapest, Hungary; 2 Wigner Research Centre for Physics, Institute for Solid State Physics and Optics, P.O. Box 49, H-1525 Budapest, Hungarybortel.gabor@wigner.hu (G.B.);; 3 Department of Atomic Physics, Institute of Physics, Budapest University of Technology and Economics, Műegyetem Rakpart 3, H-1111 Budapest, Hungary; 4 Institute of Technical Physics and Materials Science, Centre for Energy Research, P.O. Box 49, H-1525 Budapest, Hungary; czigany.zsolt@ek-cer.hu; 5 Department of Inorganic Chemistry, Institute of Chemistry, ELTE Eötvös Loránd University, Pázmány Péter sétány 1, H-1117 Budapest, Hungary; 6 Stavropoulos Center for Complex Quantum Matter, Department of Physics, University of Notre Dame, South Bend, IN 46556, USA

**Keywords:** silicon carbide, nanoparticles, functionalization, BSA, bioimaging

## Abstract

Silicon carbide nanoparticles (SiC NPs) are promising inorganic molecular-sized fluorescent biomarkers. It is imperative to develop methods to functionalize SiC NPs for certain biological applications. One possible route is to form amino groups on the surface, which can be readily used to attach target biomolecules. Here, we report direct amino-termination of aqueous SiC NPs. We demonstrate the applicability of the amino-terminated SiC NPs by attaching bovine serum albumin as a model for functionalization. We monitor the optical properties of the SiC NPs in this process and find that the fluorescence intensity is very sensitive to surface termination. Our finding may have implications for a few nanometers sized SiC NPs containing paramagnetic color centers with optically read electron spins.

## 1. Introduction

Silicon carbide (SiC) is a well-known inorganic indirect wide band-gap compound semiconductor, and it shows polytypism [1]. SiC materials can be produced by using different methods. By applying chemical vapor deposition (CVD), high-purity single crystal SiC layers can be grown from high-purity gases, such as silane and hydrocarbon precursor gases [2], where typically hexagonal polytype (e.g., 4H-SiC or 6H-SiC) layers are produced. SiC nanostructures can be synthesized with various methods. For instance, a synthesis of highly spherical cubic (3C) SiC nanocrystals (50–150 nm) using nonthermal plasma has been recently reported where the Si seed remained present in the middle of the SiC nanocrystals [3]. The vapor-liquid-solid (VLS) is also known technique to grow SiC [4]. Furthermore, there are also examples of combustion synthesis from its elements like the self-propagating high-temperature synthesis (SHS) and volume combustion synthesis (VCS) methods [5]. The synthesis temperature and additives play an important role in the formation of the polytypes, and micrometer- or few-hundred-nanometer-sized SiC nanostructures can be produced by these techniques. It has been shown, for instance, that nanowires from cubic SiC can be grown by a combustion synthesis route with using a Si/PTFE system [6].

An important feature of SiC is its high degree of inertness which makes this material promising in biological applications, e.g., SiC-related bone prosthetics and other implants [7,8,9], sensors, and quantum technology applications [10,11]. The nanocrystalline form of SiC has also been attested in various biological systems [12,13,14,15]. SiC nanoparticles (NPs) below 5 nm showed low toxicity with surface-dependent metabolic activity of monocytic cells [12]. The applicability of the SiC NPs in two-photon microscopy was also demonstrated in vitro in neuron cells [16]. Its stability, biocompatibility, and optical properties nominate SiC NPs for an auspicious building block in sensors, signal generators, and fluorescence imaging. However, for such applications, functionalization of the NPs surface is essential to give selectivity or further conjugation with other molecules, including biomolecules. Despite the encouraging results with SiC NPs, functionalization of the nanoparticles for the standard labeling protocols has not been reported yet. Despite the presence of the carboxylic groups on the surface of the as-prepared SiC NPs, the variety of the surface functional groups without further modification makes the development of a standard labeling procedure difficult. Surface modification of the ultrasmall SiC NPs leads to a change in their optical properties, and a method that keeps SiC NPs fluorescent after labeling is essential. One of the most used functional groups for conjugation is the amine group which can be targeted with many different reactions in mild conditions, which is necessary to keep the functionality of the conjugated biomolecules. The presence of complex functional groups, such as the carboxylic group on the surface of SiC NPs, makes direct functionalization even more difficult because the carbon atom of these groups cannot be counted in the crystalline lattice.

Here, we present a direct amination reaction for the synthesis of amine terminated cubic SiC NPs (SiC-NH_2_) that are suitable for standard labeling protocols [17,18,19,20]. The intramolecular hydrogen bonding related quenching of the amino groups makes SiC-NH_2_ particles dark with a recovering emission after further modification of the amino groups, i.e., in biomolecule labeling, that can reduce the background noise for certain applications while opening a door for sensitive biosensing. When the biomolecule is connected, the peptide bond (secondary amide) is formed from the NH_2_ group, which changes its optical properties. It is possible to produce C-terminal conjugates with NH_2_ termination because the COOH group of the peptide will react to the NH_2_. With this modification, the nanoparticles can connect to C-terminal peptides, and the conjugates thus obtained can bind to N-terminal receptors in the body. Therefore, it is possible to target other receptors, not only those to which the COOH end could connect. Together with known quantum bits in SiC [21], this phenomenon enables quantum sensing as well.

SiC NPs may be prepared in various ways. Some of them have been very recently reported [22,23,24,25,26,27]. Our group optimized the wet chemical etching method [26], which re ults in 1-4 nm cubic SiC NPs. The as-prepared SiC NPs are typically covered by carboxyl groups, hydroxyl groups, and other oxygen-related surface groups [28,29,30]. Previously, we synthesized amino-terminated SiC NPs with hydrosilanization of the OH-terminated SiC NPs [12]. The reaction of an -OH surface group (whether it is carboxylic, hydroxyl or inorganic) and an oxysilane creates an ether-like bond in a condensation reaction. On one hand, this is a very straightforward reaction offering a vast variety of functionalization, but on the other hand, the formed oxygen bridge often has limited stability while together with the organic molecule used to attach the functional group to the silicon atom creates a spacer between the NP core and the functional group, which is likely to eliminate the sensitivity of the nanoparticle. We note that nitrile-containing radicals were added to the surface of larger SiC particles (30–50 nm). The nitrile group can be hydrolyzed to the carboxylic group or reduced to amino group [31]. In this case, the alkyl groups also influence the SiC nanoparticle’s properties, e.g., colloidal stability [31]. Substitution of the oxygen-containing functional groups with nitrogen-containing moieties is likely to make the NPs sensitive for further conjugation as the amine surface group suffer significant changes in the conjugation reactions that modifies the optical properties of the nanoparticle.

The Hofmann degradation reaction can transform most of the oxygen-related species to amine groups, while carbon atoms that are not part of the SiC crystal, such as the C atom in the carboxyl group, can be eliminated. We applied the Hofmann degradation reaction for direct amination of the SiC NPs. The steric hindrance is very high, but the results show that the reaction is feasible [25]. The highly oxidative reaction not just transformed the surface carboxylic groups to amino groups but eliminated most of the oxide related surface groups resulting in new nitrogen-terminated SiC NPs with reaction-sensitive optical properties.

## 2. Materials and Methods

**Scheme 1:**
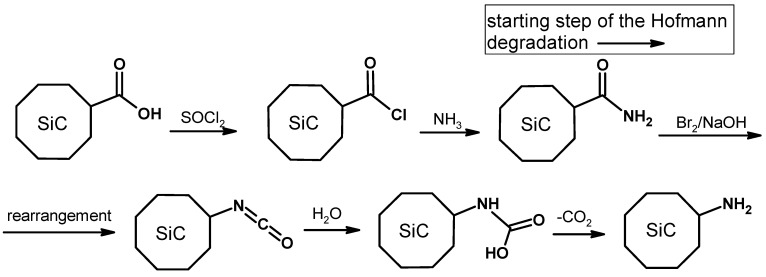
The sequence of reaction steps to create amino-terminated SiC NPs covered by carboxyl groups in water. The Hofmann degradation process starts at step 3 after the amide has formed in the previous steps.

We synthesized microcrystalline 3C-SiC (SiC-MC) in our laboratory according to the previous prescription [32]. The SiC-MC was etched to prepare SiC NPs as explained above that we call as-prepared SiC NPs in this context. The Hofmann degradation was carried out on these as-prepared SiC NPs. The reaction steps are depicted in Scheme 1. The Hofmann degradation, under ideal conditions, is the organic reaction of a primary amide to a primary amine with one fewer carbon atom. The amide is formed after the NPs are chlorinated and treated with ammonia. The sodium hypobromite in the next step transforms the primary amide into an intermediate isocyanate which is then hydrolyzed to a primary amine while ejecting carbon dioxide. We note again that many hydroxyl groups are also present on the surface of SiC NPs. The chlorination step in the process substitutes -OH groups to -Cl groups, and Cl groups are exchanged to -NH_2_ groups in the presence of ammonia. The Br_2_ molecule, the alkali solution, and the hypobromite can also induce a series of complex reactions, which we will discuss later.

### 2.1. Preparation of NH_2_-Terminated SiC NPs

SiC NPs were synthesized by the previously reported method [26]. The as-prepared aqueous solution of SiC NPs (20 mL) was entirely dried in vacuum at 50 °C. SOCl_2_ (20 mL, Merk KGaA, Germany, ReagentPlus) was added to the sample (13–18 mg). The mixture was sonicated at 80 kHz at room temperature for 1 h and at 37 kHz at 50 °C for 2 h. The SOCl_2_ excess was removed by vacuum evaporation at 40 °C. It is a crucial step to completely remove SOCl_2_ because it can precipitate when reacted with NH_3_ in the next step to create various solid products which can encapsulate SiC NPs. After the removal of SOCl_2_, 0.1 M NH_3_/dioxane solution (Merk KGaA, Darmstadt, Germany) was added. This step was followed by an ultrasonic treatment with the conditions above. The solvent was removed in a vacuum at 40 °C. The dry sample was sonicated in water for the Hofmann degradation (at 80 kHz for 1 h and at 37 kHz for 1 h); 0.5 g NaOH (VWR International, LLC, AnalR NORMAPUR, Vienna, Austria) and 0.35 mL Br_2_ (Across Organics, Geel, Belgium) were added, and the mixture was heated to 80 °C for 10 min. At the end of the reaction, the NaOBr excess was eliminated by cc. NH_3_ solution (VWR International, LLC, 32% GPR RECTAPUR). The NH_3_ excess was removed in a vacuum at 50 °C, and the pH was neutralized by H_2_SO_4_ (VWR International, LLC, AnalR NORMAPUR). The solution was concentrated from 20 mL to 3–5 mL, and size exclusion chromatography was used for product isolation. We note that these SiC NPs are not aggregated. The stationary phase was Sephadex gel (Merk KGaA, Darmstadt, Germany—Cytiva G-25), and the eluent was water.

### 2.2. Bovine Serum Albumin Conjugation

Some 4 mg of 1-ethily-3-carbodiimide hydrochloride (EDC-HCL) and 10 mg of N hydroxysulfosuccinimide (Sulfo-NHS) were dissolved in 5 mL of 1.2 mg/mL (ca. 0.1 mM) SiC-NH_2_ colloid solution after the pH was set to 5.8. Then, 5 mg of bovine serum albumin (BSA) was added to the solution. The reaction mixture was kept at room temperature for an hour. 

It was occasionally shaken gently, then centrifuged at 8500 rpm for 5 min (Heraeus Megafuge 8, Geel, Belgium), and washed with deionized (DI) water by using a 30 kDa Pall Macrosep centrifuge filter.

### 2.3. Analytical Methods

The atomic force microscopy (AFM) images were prepared by applying Bruker Dimension Icon with Bruker MPP-111, 00-10 (Leipzig, Germany) probe equipment in tapping mode. The as-prepared and NH_2_ terminated SiC NPs samples of 10 µL were diluted 100 fold in water before AFM measurements. The samples were drop-cast on a Si slide with the size of 0.5 cm × 0.5 cm, sucked by a pipette after 15 min, and finally dried at room temperature. In 3–5 selected points, a 25 µm^2^ area was scanned, and the height of at least 200 particles was measured for size distribution.

The dynamic light scattering (DLS) and zeta potential measurements were carried out with Malvern instruments (Malvern Zetasizer Nano S for DLS and Nano Z for zeta potential measurements, Cambridge, United Kingdom) in an aqueous medium by using the built-in 633 nm He-Ne laser (4 mW). The samples were placed in capillary cells (DTS1070) for DLS. We carried out 5–7 scans to obtain the DLS spectrum for a single record of measurements, where each scan lasted for 75 s. We repeated these measurements thrice with five seconds pause between the recordings for each sample. Samples were filtered through a 0.1 μm syringe filter and tempered to 25 °C before measurement in all cases. The zeta potential values were recorded from 3 measurements and each measurement consisted of 45 runs. The samples were in a 10 mm polystyrene cuvette equipped with palladium electrodes which were used for zeta potential measurements and in capillary cells (DTS1070). The pH was set with H_2_SO_4_ and NaOH solution.

Agilent Cary 4000 spectrophotometer was used to characterize the optical absorption properties of the nanoparticles. The samples were analyzed in a 10 mm quartz cuvette (Hellma 110-QS).

The Fourier transform infrared spectroscopy (FTIR) experiments were carried out on a Bruker Tensor 37 FTIR spectrometer. This method is sensitive to the chemical groups at the SiC particle surfaces [28]. Every sample was evaporated onto a Si substrate, and the spectra were measured in transmission. Absorption was calculated by the simple formula A = −log T and background subtracted, including the correction for multiple reflections in the substrate.

The X-ray photoelectron spectroscopy (XPS) measurements were carried out using a twin anode X-ray source (Thermo Fisher Scientific, Waltham, MA, USA, XR4) and a hemispherical energy analyzer with a nine-channel multi-channeltron detector (SPECSGROUP, Berlin, Germany, Phoibos 150 MCD). The base pressure of the analysis chamber was around 2 × 10^−9^ mbar. Samples were analyzed using a Mg Kα (1253.6 eV) anode without monochromatization. The SiC NP samples were dried and drop-cast onto a niobium substrate.

Photoluminescence spectroscopy (PL) measurements were performed by a Horiba Jobin-Yvon NanoLog FL3-2iHR spectrophotometer equipped with a 450 W Xenon lamp, an iHR-320 grating monochromator, and an R928P photomultiplier tube (Viena, Austria). The samples were placed in a 10 mm quartz cuvette (Hellma 110-QS, Jena, Germany).

The samples were also studied by nuclear magnetic resonance (NMR) using a Bruker Avance Neo spectrometer (400 MHz, Leipzig, Germany). The ^1^H NMR measurements were performed in DMSO-d6 to inhibit NH_2_ proton exchange. The samples were evaporated from an aqueous medium at 50 °C in nitrogen flow and sonicated in DMSO-d6.

Scanning electron microscopy (SEM) images and energy-dispersive spectroscopic (EDS) data were recorded by a TESCAN MIRA3 electron microscope equipped with an Element EDS system. The SEM images were taken by applying In-Beam Secondary Electron (SE) mode. The SiC NPs were diluted in water and drop-cast on Si substrate for the measurements.

The high-resolution transmission electron microscopy (HRTEM) images were taken in a Themis (Thermo Fisher) TEM operated at 200 kV and equipped with Cs correction in the imaging system (spatial resolution in HRTEM mode 0.8 Å). Images were recorded by a 4 k × 4 k Ceta camera using Velox software (Thermo Fischer). Aqueous suspension of SiC-NH_2_ was dropped onto a TEM grid (TED Pella) covered with an ultrathin carbon layer supported by lacey carbon after the purification.

X-ray diffraction measurements were carried using a Huber G670 Guinier Imaging Plate Camera (Rimsting, Germany) with Cu Kα radiation (1.54 Å). The values for 2θ were varied from 10° to 100°. Samples were enclosed in a glass capillary of 0.5 mm diameter and 0.01 mm wall thickness.

Raman measurements were carried out with a Renishaw InVia confocal Raman microscope (Wotton-under-Edge, UK) and a 2 W continuous 532 nm laser at 1% intensity; the power at the sample is estimated to be around 20 mW.

## 3. Results

**Figure 1:**
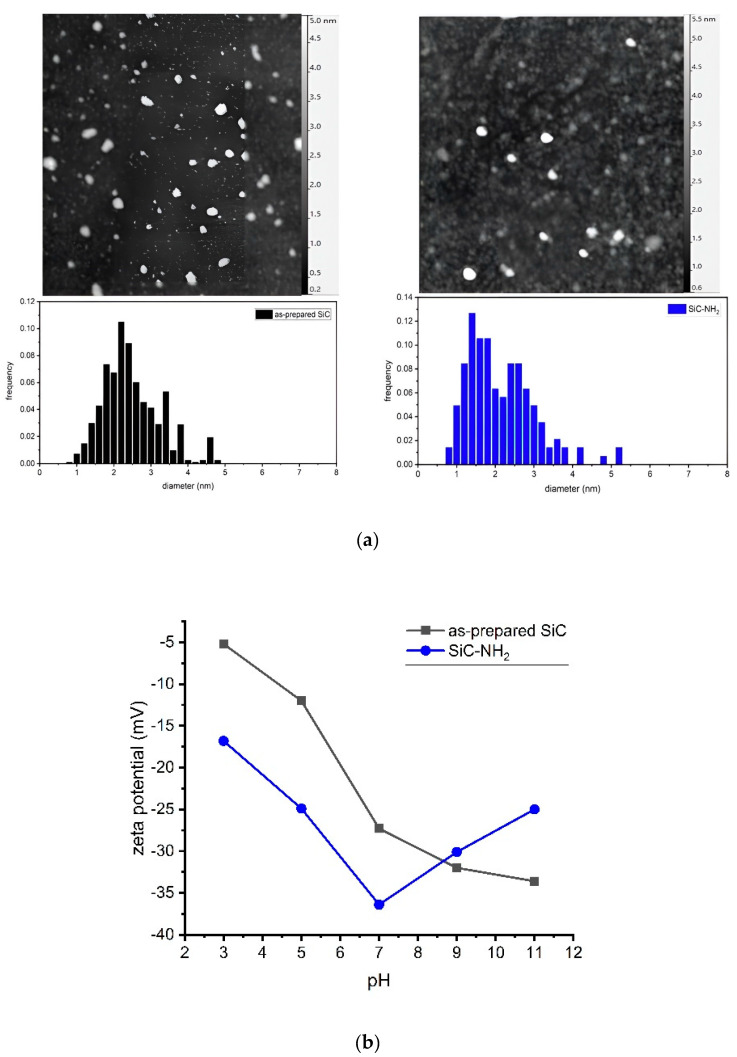
(**a**) The AFM images and size distribution of SiC NPs. The size of the images is 1 µm × 1 µm, which was selected for the size distribution analysis. (**b**) Zeta potential of the as-prepared and amino-terminated SiC NPs.

The as-prepared SiC NPs were treated as given in Scheme 1. We compared the size-distribution of the as-prepared and the amino-terminated NPs by DLS and AFM methods as shown in Figure 1a. The DLS measurement showed NPs in the size ranges of 1–4 nm with a mean size of 3 nm, and 2–8 nm with a mean size of 4 nm for the as-prepared and amine terminated samples, respectively. The larger size is due to the hydrodynamic diameter. AFM measurement gives a similar size distribution with mean sizes of 2.4 nm and 2.2 nm, and lognormal fittings provide the shape parameter σ at 0.71 and 1.04 for the as-prepared and the amino terminated samples, respectively.

The zeta potential values as a function of pH are plotted in Figure 1b. Both samples show negative values for the whole measured pH range. The plot does not show an isoelectric point which suggests stability of the colloid samples [33]. We note that the NH_2_-terminated silicon dioxide NPs with OH groups on the surface show also negative zeta potential values [34] which is very similar to our system. The OH groups are also protic groups in acid-base equilibrium on the surface [35], and the OH-terminated surface can provide more negative value than the COOH-terminated one [36].

**Figure 2:**
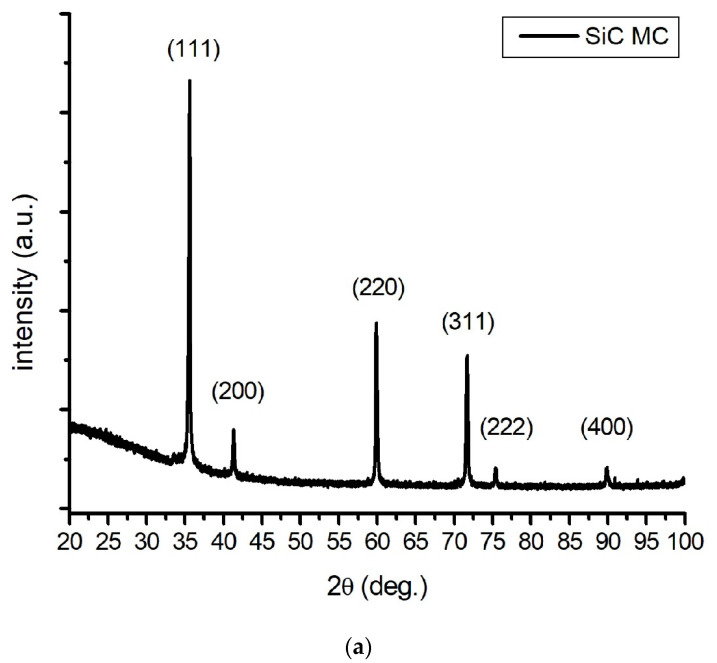

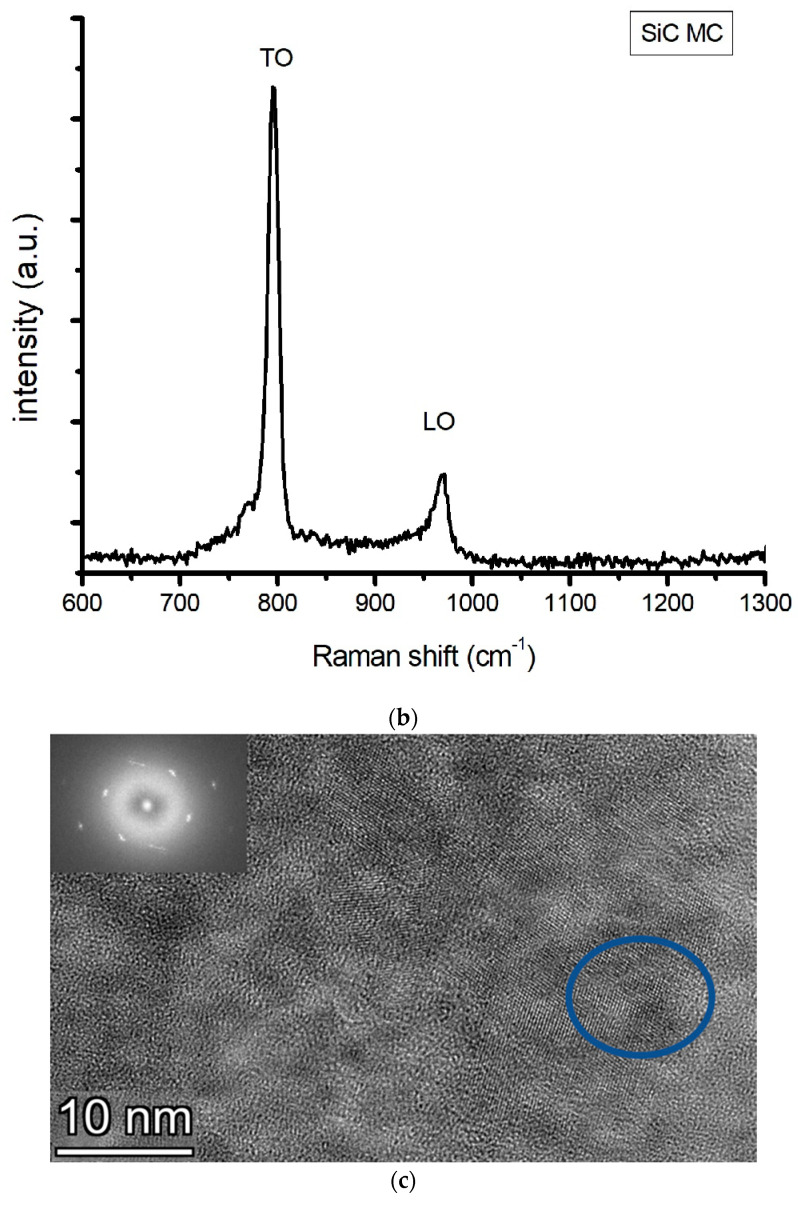

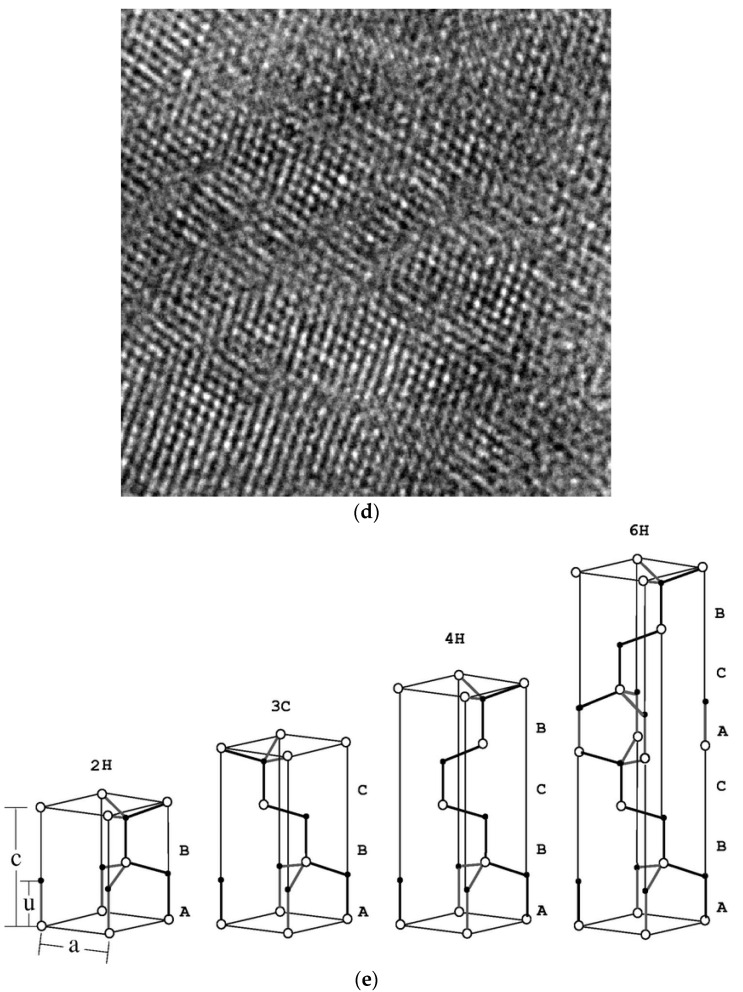
(**a**) The diffractogram of SiC-MC. (**b**) Raman measurement of SiC-MC. (**c**) HRTEM image with Fast Fourier transform (FFT) on SiC NPs after Hofmann reaction. The blue circle shows an area that is magnified in the following image. (**d**) The magnified HRTEM image is 10 nm × 10 nm. (**e**) Structure of the different SiC polytypes (black balls: carbon, white balls: silicon). 3C-SiC refers to the cubic polytype. The image is taken from Ref. [37].

The crystalline structure and presence of SiC were confirmed by XRD and HRTEM measurements. Figure 2a shows the diffractogram of SiC-MC from which we produced the as-prepared SiC NPs. The reflections clearly show that the starting material was a cubic SiC as shown in Figure 2e from which the as-prepared SiC NPs were produced. This is clearly supported by the Raman measurements too in Figure 2b which shows the longitudinal optical (LO) and transversal optical (TO) Raman modes of the cubic SiC crystal. These nanocrystals after the Hofmann degradation process were further studied by HRTEM which resulted in the FFT image shown in the inset of Figure 2). Four periodically repeated distances were extracted from the FFT of the HRTEM image that correspond to the lattice spacings of 2.52 Å (111), 2.18 Å (200), 1.54 Å (220), and 1.26 Å (222), respectively, where the respective planes are given in the parentheses. These lattice spacings are characteristic for cubic SiC. The SiC crystal structure may be also recognized in the enlarged view of the HRTEM image in Figure 2d. It proves that the chemical reactions do not damage the crystalline structure in the core of the SiC NPs.

**Figure 3:**
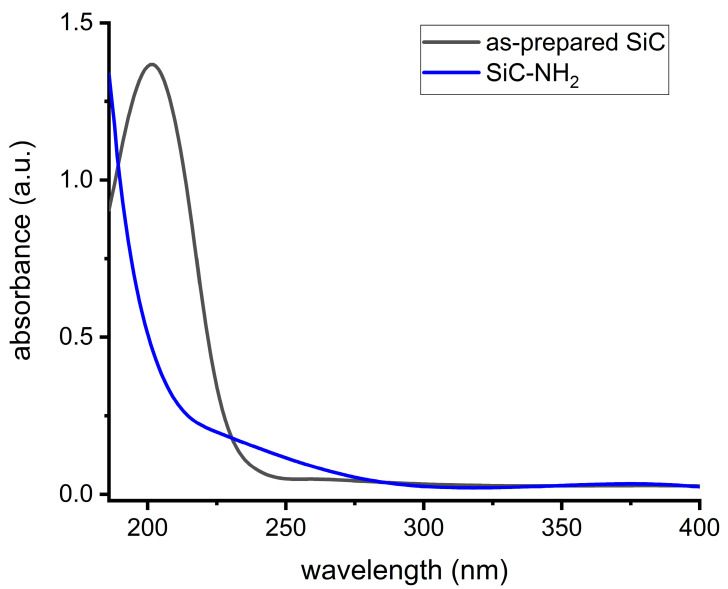
UV–VIS spectra of as-prepared and amino-terminated SiC NPs.

The transformation from carboxyl to amine termination is also reflected in the UV–VIS spectra (see Figure 3). The as-prepared material exhibits the typical -COOH absorption at ~200 nm that disappears in the amine terminated sample. The lack of peaks in the latter proves that no other chromophores are present on the surface of the NPs.

**Figure 4:**
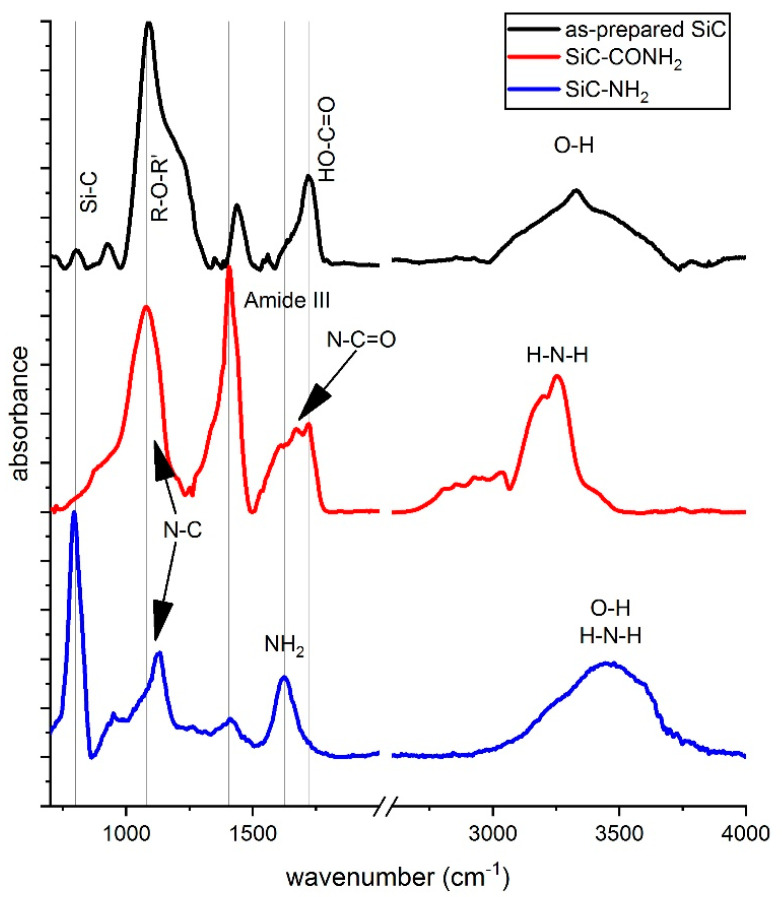
FTIR absorbance spectra of as-prepared SiC NPs, amide intermediates (SiC-CONH_2_), and the amino-terminated SiC NPs (SiC-NH_2_) from the Hofmann degradation. Spectra were vertically shifted for the sake of clarity.

The FTIR results are shown in Figure 4. In particular, we recorded the spectrum of as-prepared SiC NPs (as-prepared material), the samples after accomplishing the first two steps in Scheme 1 (SiC-CONH_2_ material), and the final step in Scheme 1 (SiC-NH_2_ material). In the FTIR spectrum of the as-prepared material, the very broad feature between 3000 and 3700 cm^−1^ is associated with the various forms of -OH groups, whereas the strong C=O stretching vibration of carboxyl groups appears at 1720 cm^−1^ [28]. The different “oxygen bridge” vibrations (Si-O-Si, Si-O-C, C-O-C) are around 1100 cm^−1^. The band at 1435 cm^−1^ can be assigned to either the OH- or CH-bending modes [38].

For the SiC-CONH_2_ material, a radical change in the FTIR spectrum was observed. The very broad band above 3000 cm^−1^ is replaced by a narrower band which is a signature of the -OH group elimination. The peak maxima are at 3196 and 3253 cm^−1^, typical of the amide A band from symmetric and asymmetric vibrations of the amide NH_2_. The new weak bands in the 2700–3000 cm^−1^ region might come from C-H and N-H vibrations from the amine salts. The triplet peak at 1600 and 1670 cm^−1^ may be associated with -NH_2_ of the amide, and the 1720 cm^−1^ band is associated with the remaining carbonyl groups. The strong band at 1404 cm^−1^ is the amide III, a typical C-N stretching mode. The bands associated with the surface Si-O and C-O vibrations become narrower. The features in the region of 1000–1142 cm^−1^ might be partially associated with C-O and C-N stretching modes.

For the SiC-NH_2_ material, the broad feature is associated with the -OH groups from the surface, adsorbed water, and the stretching vibrations of NH_2._ The intensive new feature at about 1621 cm^−1^ is related to the deformation vibration mode of the primary amino group, whereas the amide II, amide III bands, and the carboxyl band disappeared almost completely. Another feature at 1132 cm^−1^ might be associated with the torsion vibration mode of the C-N bond [39]. Together with the appearance of the C-N and N-H related vibrations, the SiC-NH_2_ material shows a strong reduction in the intensity of Si-O and C-O vibration-related bands [40,41,42,43]. The intense peak in the product at 800 cm^−1^ is attributed to SiC which becomes visible after removing the oxide and organic groups at the surface of SiC NPs by the Hofmann degradation process. This feature in the FTIR spectrum of SiC-NH_2_ NPs is in good agreement with a previous FTIR study of one-dimensional SiC nanostructures [44].

Besides FTIR, the XPS method was used to study chemical bonds of the SiC NPs. We also calculated the atomic concentration of the elements from the XPS survey spectra that is compared to the EDS results (Table 1). It can be observed that silicon, oxygen, and nitrogen concentrations decrease. The as-prepared samples might contain nitrogen impurities from the applied nitric acid for synthesis. The decrease in Si concentration is, however, surprising.

**Figure 5:**
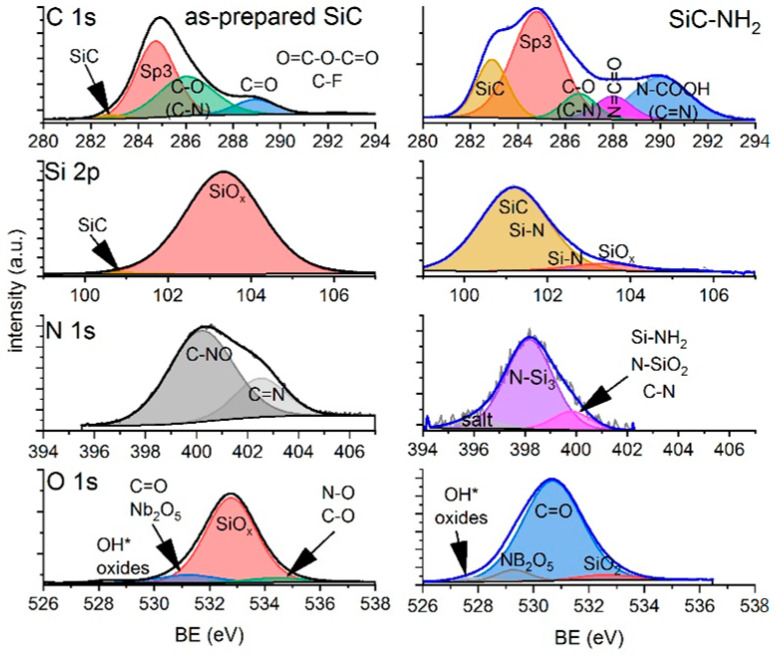
C 1s, Si 2p, N 1s, and O 1s XPS spectra of the as-prepared (**left**) and SiC-NH_2_ (**right**) NPs. Samples were drop cast onto a Nb substrate, and all spectra were corrected to the C sp^3^ peak (284.8 eV). X-ray photoemission intensity was plotted as a function of binding energy.

The high-resolution structure of the C 1s peak in the XPS spectrum shows a strong sp^3^ C signal at around 285 eV from C-O and C=O bonds and a low relative concentration of SiC (1.2%) bond (288 eV) for the as-prepared SiC NPs (see Figure 5). The SiC contribution increased to 16% in the SiC-NH_2_ NPs. The low relative concentration of SiC bond for the studied NPs is not surprising. For a 2.5 nm nanoparticle, about 80% of the atoms forming the crystal are at the surface [45] that contain various chemical bonds which significantly differ from those in the core of Si-C bonds.

The Si 2p XPS peak shows a high concentration of the Si-O bond for the as-prepared sample that is significantly reduced in the SiC-NH_2_ NPs.

The N 1s XPS peak shows a significant change during the reaction. For the as-prepared SiC NP, only C-NO and possibly C=N bonds can be seen, which is expected due to the strong acid treatment applied during the SiC NPs synthesis. After the reaction, the dominant peak around 398 eV can be associated with the N-Si_3_ bond. Other Si-N related bonds appeared, too, i.e., N-SiO*_x_* alongside the N-C bond at around 400 eV.

The elimination of the Si-O bond from the systems after Hofmann degradation is further supported by the O 1s XPS peaks, in which the O-Si signal decreased significantly after the reaction. However, the O 1s XPS peaks cannot be adequately analyzed as we also observe the oxide from the Nb substrate and impurities for which the associated O 1s XPS spectra suffer different charge related chemical shifts.

**Figure 6:**
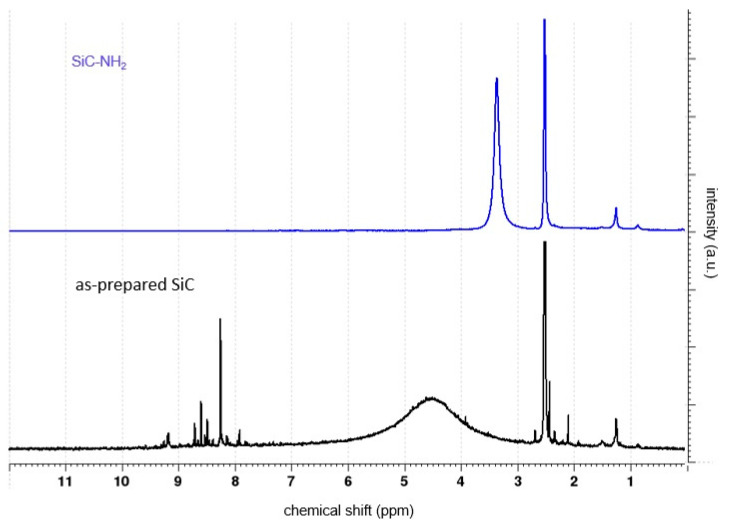
^1^H-NMR spectra of the as-prepared (black) and amino-terminated (blue) SiC NPs in deuterated-DMSO (DMSO-d6). The peak at 2.52 ppm is the signal of the solvent and the wide signal comes from the water at 4.50 ppm.

The chemical structure of the surface was further studied by the NMR method. The ^1^H NMR spectrum (see Figure 6) shows -OH signals between 2.6 and 4 ppm for the as-prepared material. The large number of signals may be explained by the presence of both Si-OH and C-OH bonds at various distances from each other.

The nearby -OH surface groups create H-bonds that can shift the -OH related ^1^H NMR signals. We associate the 8–9.2 ppm peaks with the carboxyl groups for the as-prepared SiC NPs. We note that the specific position of carboxyl groups in organic molecules is 9–11 ppm. Our argument is based on our previous findings that the carboxyl groups at the surface of SiC are proximate (about 3 Å), and many of them are attached to Si atoms [28]. The proximate carboxyl groups may interact with enhanced electron density around the H atom of the -OH part of the carboxyl group. The presence of H_2_O and silacarboxyl groups also reduces the chemical shift of the carboxylic protons [46]. For the SiC-NH_2_ NPs, the carboxyl peaks at around 8 ppm disappeared, and new peaks appeared at 3.36 ppm that can be associated with N-H signals of the protonated amines at neutral pH.

## 4. Discussion

We tested the Hofmann degradation reaction for direct amination of ultrasmall SiC NPs. FTIR and NMR analyses also show that this reaction mechanism works well on SiC, creating amine- and other nitrogen-containing groups on the surface. However, FTIR and XPS also show that the reaction is more complex than a standard Hofmann degradation process.

According to the NMR results and previous ab initio calculations [28], a significant amount of the surface carboxyl groups forms a bond with silicon at the surface of SiC NPs. The consequence is that the ideal Hofmann degradation creates C-NH_2_ and Si-NH_2_ groups on the surface of SiC NPs. Indeed, the NMR spectrum of the product shows that amines are in two different environments on the surface, and the chemical shift between them can be explained by the presence of a Si-C bond which introduces a large negative polarization towards the carbon atom, unlike the case of a homopolar C-C bond.

XPS and FTIR data show a strong decrease in the number of Si-O bonds and an increase in the relative SiC content in the SiC-NH_2_ material. EDS also shows a decrease in Si and O content after the reaction. The basic hypobromite solution applied for the Hofmann degradation by adding Br_2_ to the NaOH solution indeed has strong oxidizing capability. It is reported that hypochlorite can degrade carbon nanotubes completely [47], and the Hofmann degradation process was proven to oxidize and exfoliate graphene oxide by a continuous attack on the oxidized carbon [48]. Br_2_ is a known etchant for silicon [49], and bromide salts can break Si-O-Si bonds in amine functionalized siloxanes [41]. Due to the complexity of the studied SiC NPs system and the reaction routes in the Hofmann reaction, further studies are needed to understand the elimination of the many surface groups during the reaction, but our results highlight that the Hofmann degradation reaction applied to ultrasmall SiC NPs degrades the surface of the nanoparticles and leaves NH_2_ groups and nitrogen-terminated Si facets on the surface. The elemental analysis shows reduced oxygen content, suggesting that oxygen-containing groups are still present on the surface. We conclude from the parallel evaluation of the data that Hofmann degradation eliminated most of the original carboxylic groups from the surface as the carboxyl group related peaks disappeared from the NMR, UV–VIS and IR spectra, and the significant shifts in the XPS C and O peaks indicate that the possible C=O double bonds are in a different chemical environment. NMR and FTIR indicate N related surface groups: Si-NH_2_, C-NH_2_, and N-C-O and some newly formed C-OH and Si-OH groups, and other than those, XPS implies Si_3_N bonds. XPS and FTIR are sensitive methods for functional groups and chemical bonds, but the silent band systems in FTIR are challenging to analyze [50], and the overlapping chemical shift in XPS also makes a detailed surface mapping difficult. Nevertheless, surface analysis shows that Hofmann degradation is possible on a nanoparticle surface, and in the case of SiC NPs, the process eliminates the Si-O-Si surface groups and leads to SiC NPs terminated by NH_2_ with other moieties.

A result of replacing the surface moieties and the remaining OH groups is the quenching of the photoluminescence (PL) signal of SiC-NH_2_. It was found in the CdTe and Au NP assembly that the luminescence of the nanoparticles quenches due to the developed intermolecular hydrogen bonding in the system [51]. The same happens between the amine and OH groups on SiC NPs [37]. However, this quenching effect should be diminished when the amino group participates in a chemical reaction to form seconder amines, amides, or peptide bonds and break the hydrogen bonds responsible for the quenching effect.

**Figure 7:**
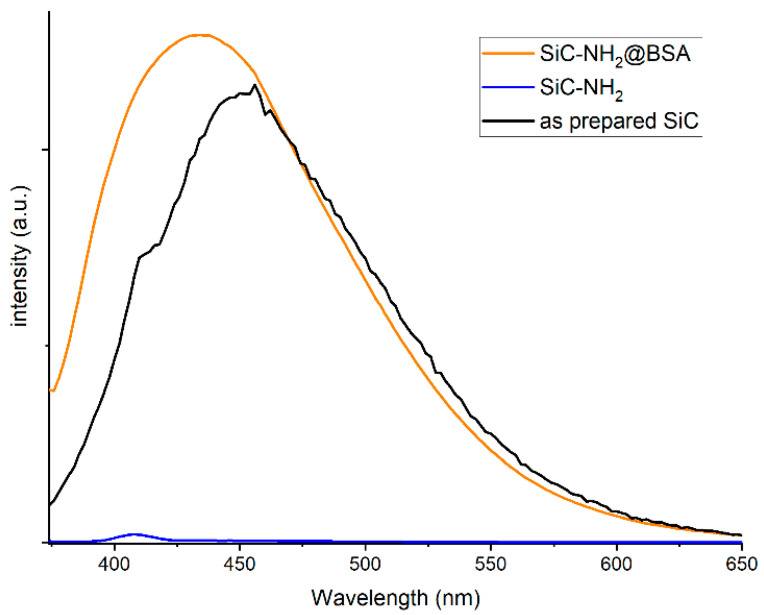
PL spectra of as-prepared SiC NPs, and SiC-NH_2_ before and after conjugation with BSA. The excitation wavelength was 360 nm.

This is the case, for example, when the standard conjugation technique is used. Cross-linking and conjugating biomolecules or other chemical substances to different substrates are widely used in biomaterials, biosensors, biochips, chromatography, and labs-on-a-chip. Carbodiimide compounds provide the most popular and versatile method for labeling or crosslinking to carboxylic acids. The EDC/NHS activation approach possesses many merits, including high conversion efficiency, mild reaction conditions, and excellent biocompatibility with little or no influence on the bioactivity of the target molecules. Due to these advantages, EDC/NHS activation of carboxylic acids and the following amidation reaction have been widely applied in biomolecular conjugation and immobilization of proteins, peptides, and DNAs. The recovery of the PL signal upon conjugation offers good sensing capability as the reference (unconjugated) signal is almost zero. In such a case, the PL intensity is proportional to the amount of conjugation, and there is no need for spectral filtering, inner standards, or other methods for signal evaluation. We used such standard conjugation protocol to link the SiC-NH_2_ nanoparticles to the Bovin Serum Albumin protein, an abundant and well documented biomolecule, which has been studied in the presence of the ultrasmall SiC NPs [52] we used for the synthesis of SiC-NH_2_. We measured the PL spectra of the SiC-NH_2_ materials and the SiC-NH_2_@BSA conjugate. As can be seen in Figure 7, the SiC-NH_2_ product material has negligible emission compared to that of the as-prepared material, but when the SiC-NH_2_ NPs are conjugated with the BSA, the emission recovers as the BSA conjugation turns amino groups to amide groups and breaks the hydrogen bonds.

The non-covalent interaction between the oxygen terminates of SiC NPs and BSA [52] causes long lasting association. As a consequence, when the NPs are separated from the protein with centrifugation, dialysis, or filtration, a slow release of SiC NPs can be detected, and the SiC NPs can be measured in the filtrate in a slowly decreasing concentration during repeated filtration (i.e., solvent exchange or washing cycles). The same trend can be seen when the as-prepared SiC NPs are used in the EDC protocol in which the carboxyl group on the surface is activated for the conjugation because the particle–particle association inhibits the conjugation reaction. This is not the case for SiC-NH_2_. We did not detect SiC by UV–VIS and PL measurement even in the first filtrate after conjugation. At the same time, mixture of BSA and SiC-NH_2_ released SiC-NH_2_ with an exponential concentration decrease during filtration suggesting no non-covalent interaction between SiC-NH_2_ and BSA.

## 5. Conclusions and Outlook

In this paper, we presented a method to prepare amino-terminated SiC NPs by applying the Hofmann degradation process. We confirmed the success of the reactions by FTIR, XPS, and NMR spectroscopy. We used an EDC linker method to prepare SiC–BSA conjugate which can be considered as a model for the functionalization of SiC NPs. We demonstrated that the amino termination quenches the fluorescence of SiC NPs. However, the SiC-NH_2_ NPs conjugated with BSA intensively fluoresce again. Thus, the functionalized SiC NPs can be applied as fluorescent biomarkers.

The direct amino-termination of SiC NP could be of importance in the doping of SiC NPs by nitrogen and in the optical properties of SiC NPs hosting color centers and qubits. Recently, we have developed 4–5 nm SiC NPs hosting these qubits [21,53]. The defect qubit should fluoresce otherwise it cannot be used for sensing. The amino-termination of such SiC NPs might also quench the luminescence from defect qubits. However, we showed that the BSA conjugated SiC-NH_2_ NPs restore the luminescence of the as-prepared SiC NPs; thus, we think it is likely that the same effect could be observed for SiC NPs hosting color centers. The defect qubit state depends on the charge state of the defect which can be influenced by doping. Nitrogen is a typical n-type dopant which helps to keep the photoexcited electrons inside the nanoparticles. The immediate link between the surface of SiC NP and the -NH_2_ group brings a hope that surface doping might be viable with this method if the -NH_2_ group can be attached to a surface vacancy of SiC NPs. These issues are the subject of future studies and out of the scope of the present study.

## Figures and Tables

**Table 1:** Measured atomic concentrations in the samples by XPS and EDS methods.

Method	XPS/EDS (at%)			
Sample	C	N	O	Si
as-prepared	30.9/22.3	2.6/2.1	36.2/26.1	16.8/8.8
SiC-CONH_2_	58.8/-	0.5/-	20.9/-	18.8
SiC-NH_2_	46.3/47.1	1.2/1.9	27.2/7.8	3.2/0

## Data Availability

All the data are available from the authors upon reasonable request.
